# Combining multi-site magnetic resonance imaging with machine learning predicts survival in pediatric brain tumors

**DOI:** 10.1038/s41598-021-96189-8

**Published:** 2021-09-23

**Authors:** James T. Grist, Stephanie Withey, Christopher Bennett, Heather E. L. Rose, Lesley MacPherson, Adam Oates, Stephen Powell, Jan Novak, Laurence Abernethy, Barry Pizer, Simon Bailey, Steven C. Clifford, Dipayan Mitra, Theodoros N. Arvanitis, Dorothee P. Auer, Shivaram Avula, Richard Grundy, Andrew C. Peet

**Affiliations:** 1 Institute of Cancer and Genomic Sciences, School of Medical and Dental Sciences, University of Birmingham, Birmingham, UK; 2 Oncology, Birmingham Women’s and Children’s NHS Foundation Trust, Birmingham, UK; 3 RRPPS, University Hospitals Birmingham NHS Foundation Trust, Birmingham, UK; 4 Radiology, Birmingham Women’s and Children’s NHS Foundation Trust, Birmingham, UK; 5 Psychology, College of Health and Life Sciences Aston University, Birmingham, UK; 6 Aston Neuroscience Institute, Aston University, Birmingham, UK; 7 Radiology, Alder Hey Children’s NHS Foundation Trust, Liverpool, UK; 8 Oncology, Alder Hey Children’s NHS Foundation Trust, Liverpool, UK; 9 Sir James Spence Institute of Child Health, Royal Victoria Infirmary, Newcastle upon Tyne, UK; 10 Wolfson Childhood Cancer Research Centre, Newcastle University Centre for Cancer, University of Newcastle, Newcastle upon Tyne, UK; 11 Neuroradiology, Royal Victoria Infirmary, Newcastle Upon Tyne, UK; 12 Institute of Digital Healthcare, WMG, University of Warwick, Coventry, UK; 13 Sir Peter Mansfield Imaging Centre, University of Nottingham Biomedical Research Centre, Nottingham, UK; 14 NIHR Nottingham Biomedical Research Centre, Nottingham, UK; 15 The Children’s Brain Tumor Research Centre, University of Nottingham, Nottingham, UK

**Keywords:** Predictive markers, Paediatric research, Tumour biomarkers, Cancer imaging

## Abstract

Brain tumors represent the highest cause of mortality in the pediatric oncological population. Diagnosis is commonly performed with magnetic resonance imaging. Survival biomarkers are challenging to identify due to the relatively low numbers of individual tumor types. 69 children with biopsy-confirmed brain tumors were recruited into this study. All participants had perfusion and diffusion weighted imaging performed at diagnosis. Imaging data were processed using conventional methods, and a Bayesian survival analysis performed. Unsupervised and supervised machine learning were performed with the survival features, to determine novel sub-groups related to survival. Sub-group analysis was undertaken to understand differences in imaging features. Survival analysis showed that a combination of diffusion and perfusion imaging were able to determine two novel sub-groups of brain tumors with different survival characteristics (p < 0.01), which were subsequently classified with high accuracy (98%) by a neural network. Analysis of high-grade tumors showed a marked difference in survival (p = 0.029) between the two clusters with high risk and low risk imaging features. This study has developed a novel model of survival for pediatric brain tumors. Tumor perfusion plays a key role in determining survival and should be considered as a high priority for future imaging protocols.

## Introduction

Brain tumors represent one of the most common causes of pediatric and adult oncological mortality. Particular challenges are faced in clinical pediatric oncology research due to the highly heterogeneous nature of pediatric tumors, combined with the relative rarity of the disease in the general population^1^. Despite this, multi-center studies have allowed impressive advances to be made in the understanding of the major types of children’s brain tumors and these are starting to change clinical practice^2,3^. The majority of studies have relied on analysis of tumor tissue; however, medical imaging is becoming increasingly able to probe tissue properties and has the advantage that measurements are made directly in vivo. This is particularly important for probing the tissue microenvironment since quantities such as perfusion cannot be readily determined in tissue samples. Imaging therefore has the potential to provide new biomarkers of prognosis which can be obtained early and throughout the patient journey.

Recently, an increased understanding of pediatric brain tumor biology has enabled more accurate prognostication for individual patients. The findings have largely been based on molecular genetic markers identified in tissue. For example, in medulloblastoma, biological subgrouping has shown that WNT subgroup tumors have an excellent prognosis whereas group 3 tumors and subsets of SHH tumors have an inferior outcome^4^. However, in even rarer tumors, such as atypical rhabdoid tumors (ATRT), or midline gliomas, where biopsy derived tissue is challenging to acquire, it is more difficult to perform molecular subtyping studies^5^. Therefore, clinical studies have been more difficult to perform in meaningful numbers for many such rare tumor types, and the small biopsies taken may not provide a representative view of the tumor, particularly its microenvironment.

Medical imaging is an important diagnostic aid for brain tumors, since it is non-invasive and can include the whole tumor and surrounding tissue. It is also capable of probing the tumor microenvironment in vivo, improving our understanding of the in vivo neovascularization and cellularity of the tumor, as well as surrounding cerebral tissue through perfusion and diffusion imaging, respectively^6,7^. However, as mentioned above for biological studies, recruiting large numbers of patients for imaging studies is challenging, and often requires large multi-center trials to glean meaningful results. In spite of this, these non-invasive modalities represent highly attractive methods to derive crucial information surrounding the diagnosis and progression of tumors.

Diffusion imaging is available on every major commercial MRI scanner and is routinely used to assess brain tumors^8^. Apparent diffusion coefficient (ADC) maps represent the speed of water motion in the tissue and this correlated cellularity. Perfusion imaging is often acquired either with dynamic susceptibility contrast (DSC) or arterial spin labelling (ASL) techniques^9,10^. DSC imaging is undertaken through the introduction of an exogeneous contrast agent containing gadolinium, and the passage of this bolus through the cerebral vasculature is rapidly imaged and post-processed to form quantitative cerebral blood volume and flow maps^11^. ASL is an approach for measuring cerebral perfusion, harnessing the use of radiofrequency tagging of blood in the supplying vessels to create blood flow-based image contrast^12^. This technique is increasing in popularity due to the removal of the need for a gadolinium containing contrast agent bolus, which may be retained within the brain^13^.

Studies have shown that diffusion and perfusion imaging are able to discriminate between pediatric tumor types in vivo*,* with high cellularity and perfusion in high grade tumors, and vice versa for low-grade^14,15^. This data, in turn, has informed survival analysis models using traditional methods such as Cox-regression to derive significant covariates from imaging data^16^. In particular, ADC mean, elevated cerebral blood flow, and image derived texture parameters have been found to be significant factors in long-term pediatric brain tumor survival^7,17,18^. However, studies which combine these imaging techniques are lacking. Moreover, the use of machine learning has not been implemented in survival analysis of these data types, despite being so successful in improving diagnosis from molecular and imaging data.

In this study we have therefore taken a novel approach to the understanding of risk and survival in pediatric brain tumors through combining diffusion and perfusion MRI and combining this with both supervised and unsupervised machine learning to determine key imaging derived risk factors and novel prognostic groups.

## Methods

### Patient recruitment and imaging

69 participants with suspected brain tumors (medulloblastoma (N = 17), pilocytic astrocytoma (N = 22), ependymoma (considered high grade, N = 10), other tumors (N = 20) are found in Supplementary Document 1. They were recruited from four clinical sites in the United Kingdom (Study approved by regional ethics committee, ethics reference: 04/MRE04/41, Birmingham Children’s Hospital, Newcastle Royal Victoria Infirmary, Queen’s Medical Centre, Alder Hey Children’s Hospital, Liverpool). Parents/guardians of participants gave informed consent for their child to be recruited to this study. All methods were performed in accordance with the relevant guidelines and regulations. Recruitment took place been 2009 and 2017. Participants underwent MRI, protocol discussed below, before treatment. Diagnosis was confirmed on tissue samples obtained by surgery performed for clinical reasons. In summary there were 37 complete resections, 17 incomplete resections, 1 open biopsy and 10 stereotactic biopsies with information missing on 4 patients. Further treatment was undertaken according to the clinical needs of the patient as determined by the local tumor board supported by national guidelines and external opinions where appropriate. Radiotherapy tended to be used in the more aggressive tumors and older children, chemotherapy was widely used and tended to be more intensive in younger children with aggressive tumors. Since the study included many different tumor types, stages and patient ages, the treatments varied widely and was tailored to the patient’s need. The results of the study were not used in any way to determine or alter treatment. The median age of the cohort at diagnosis was 8 years (range 16 days to 17.6 years). The distribution in Chang stage adapted for use across tumor types was 39 M0, 5 M1, 9 M2, 4 M3, 12 unknown. Diagnoses were made according to the WHO Classification in use at the time of diagnosis and the cohort composition mirrors that found nationally. Tumors were assigned to high- (3&4) and low-grade (1&2) groups. The median follow-up time for the cohort was 4.4 years. Full cohort details can be found in supplementary document 1.

The imaging protocol for all participants was performed either at 3 or 1.5 T and included standard anatomical imaging (T_1_-weighted pre- and post-contrast and T_2_-weighted) as well as diffusion and dynamic susceptibility contrast imaging covering the tumor volume (imaging sequence details found in Supplementary Table 1). Additional clinical data (age at diagnosis and gender) were also collected for analysis. During the data acquisition period a national protocol was in place for both conventional MRI and the advanced imaging analyzed here, some variability in acquisition sequence and parameters was allowed within the protocol^19^.

### Image post-processing and analysis

Apparent diffusion coefficient (ADC) maps were calculated from diffusion weighted imaging (DWI) using a linear fit between the two b-value images in Matlab (The Mathworks, MA, 2018a), voxels were fit after the exclusion of noise voxels (any voxel less than a signal to noise ratio of 5 was excluded). DSC time-course data were processed using conventional methods to provide uncorrected cerebral blood volume (uCBV) maps, with a leakage correction undertaken to produce corrected cerebral blood volume (cCBV) and K2 maps^20^.

T_2_-weighted imaging and ADC maps were registered to the first DSC volume with SPM12 (UCL), using a non-linear spatial transform with mutual information. Regions of interest (ROI) segmenting the tumor volume were drawn on the T_2_-weighted imaging^21^ after viewing the whole available image set, large cysts and peri-tumoral oedema were excluded.

Image analysis was performed in Matlab (2018b, The Mathworks, MA), with the image mean, standard deviation, skewness, and kurtosis were calculated on a volume by volume basis for ADC and UCBV/CCBV/K2 maps for regions of interest and the whole brain as previously described^21^. Briefly, a binary whole brain mask calculated and eroded by 3 voxels to remove any signals outside of the head. The whole brain mask included the tumor. Tumor volume (cm^3^) was calculated from the T_2_ ROI masks drawn by S.W. (A clinical physicist with more than 15 years of experience in neuroimaging and trained by a consultant pediatric radiologist with 17 years experience L.M., Grist et al.^21^), a random sample of 17 cases had ROIs reviewed by A.O. a consultant pediatric radiologist with 7 years of experience. The list of imaging features are found in the supplementary materials. Regions of interest were also drawn in normal appearing deep grey and white matter for each participant to calculate average diffusion and perfusion measures in normal appearing tissue by J.G. (A physicist with more than 5 years of experience in neuroimaging). Each ROI contained at least 50 voxels. Medulloblastoma Chang stage was derived from radiological reports and lumbar puncture fluid information, other tumors were given a stage using an equivalent definition.

### Histological and genetic analysis

Histological (including MiB1, Ki67, Glial fibrillary acidic protein (GFAP), INI-1, Isocitrate dehydrogenase-1 (IDH-1), Neuron specific enolase (NSE), S-100, BAF47, BRAF fusions, P53) and genetic data (MYC status and medulloblastoma sub-type), where available, were collected from local sites and are found in Supplementary Document 1.

Medulloblastomas were analyzed for histological type, subgroup, and MYC and MYCN amplification status were determined by protocols established at Newcastle University^22–24^. Medulloblastoma histology was centrally reviewed at the Royal Victoria Infirmary. Data are summarized in Supplementary Document 1.

### Statistical analysis

All statistical analyses were performed in R (3.6.1) with significance defined at p < 0.05, and Bonferroni correction for multiple comparisons used where appropriate.

**Figure 1:**
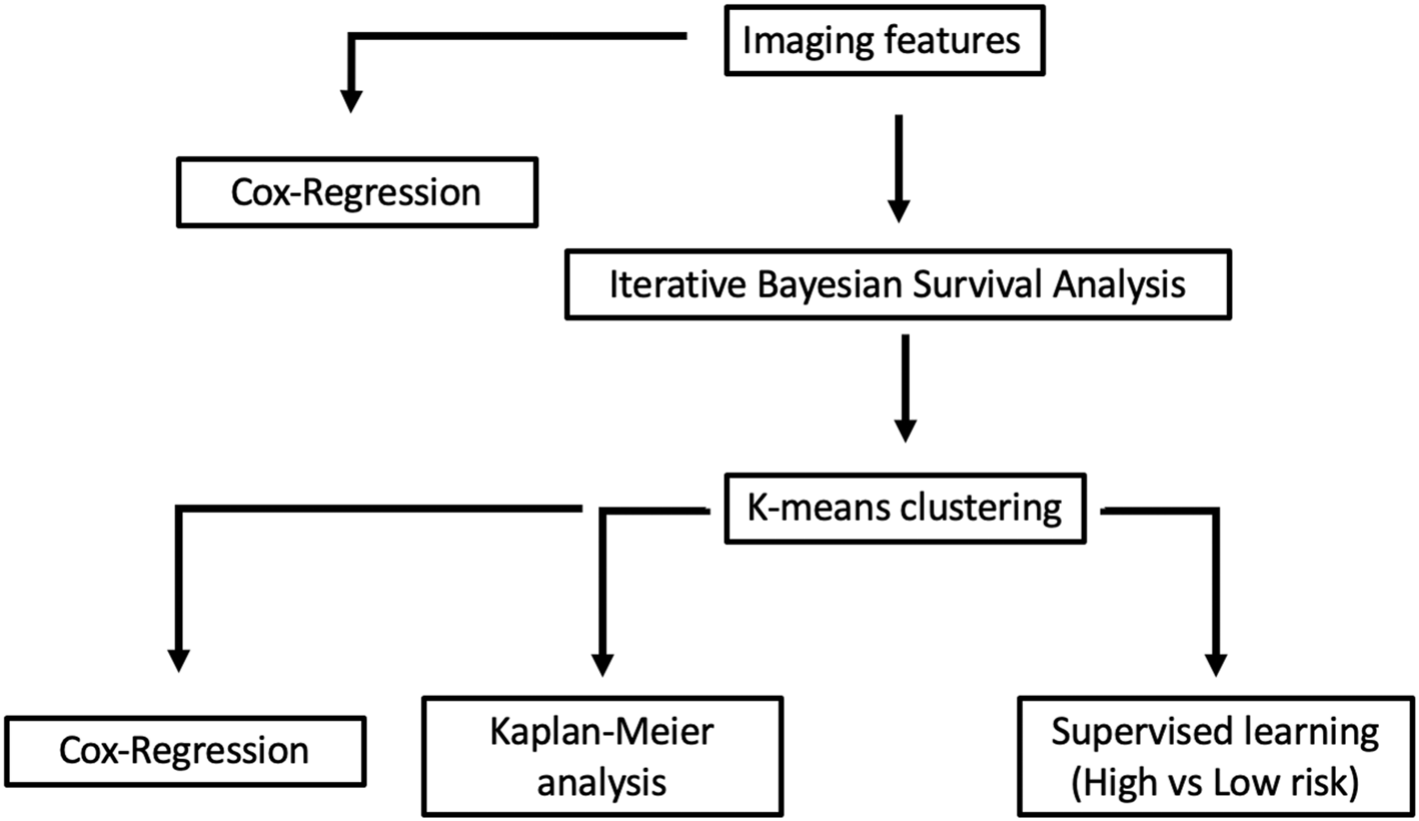
Data processing pipeline used in this study.

The data processing pipeline used in this study is summarized in Fig. 1.

### Univariate statistical analysis

Data normality was assessed using a Shapiro–Wilk test. Subsequently, differences in clinical and imaging features between high- and low-grade tumors were assessed using unpaired t-tests or Mann–Whitney *U* tests, where appropriate. Area under the ROC curve (AUC) values were calculated for each imaging feature for high/low grade discrimination. Differences in high-/low- risk (defined below) participants were assessed using unpaired two-tailed t-tests or Mann–Whitney *U* test, depending on data normality. A chi squared test was performed to assess for differences in cohort characteristics including surgical resection rates between high and low risk clusters (described below).

After unsupervised clustering (described below), further Mann–Whitney *U* tests were performed to assess for differences in imaging features between low grade tumors in low and high-risk categories, and between alive high-grade tumors in low- and high-risk categories.

### Survival and correlation analysis

Univariate Cox-regression was performed with each individual imaging feature, clinical data, and tumor grade used to assess survival hazard coefficients. Tumor grade and type were not used in the analysis detailed below.

Iterative Bayesian survival analysis was undertaken using the iterative BMAsurv package in R using fivefold stratified cross validation to determine the posterior probabilities and coefficients of the top 5 imaging features that best describe the survival data^25^. Iterative analysis including up to 15 data features in combination at any one time.

### Unsupervised and supervised machine learning

K means clustering was performed with the imaging features from Bayesian survival analysis, with the optimal number of clusters determined from the largest average silhouette width. Groups were clustered into high and low risk groups, and subsequently used for further Kaplan–Meier analysis to assess for differences in survival between clusters.

Supervised machine learning using the aforementioned Bayesian features was used to predict high/low risk groupings using the Orange toolbox (Orange) in Python (3.6), with Random Forest, a single layer Neural Network, and a support vector machine used. Validation of classifiers was performed using tenfold stratified cross-validation.

Clinical and imaging data were subset into Whole Brain (WB), and Region of Interest (ROI) features, and tumor volume and used for supervised learning. Principal component analysis was used to reduce data dimensionality with 95% of data variance or N-1 (where N is the size of the smallest group) used. The top 5 Bayesian features were also used as input into the classifiers, with no further principal component analysis performed. Classifier performance was determined from the classifier accuracy (% correctly classified cases) and F-statistic.

## Results

**Figure 2:**
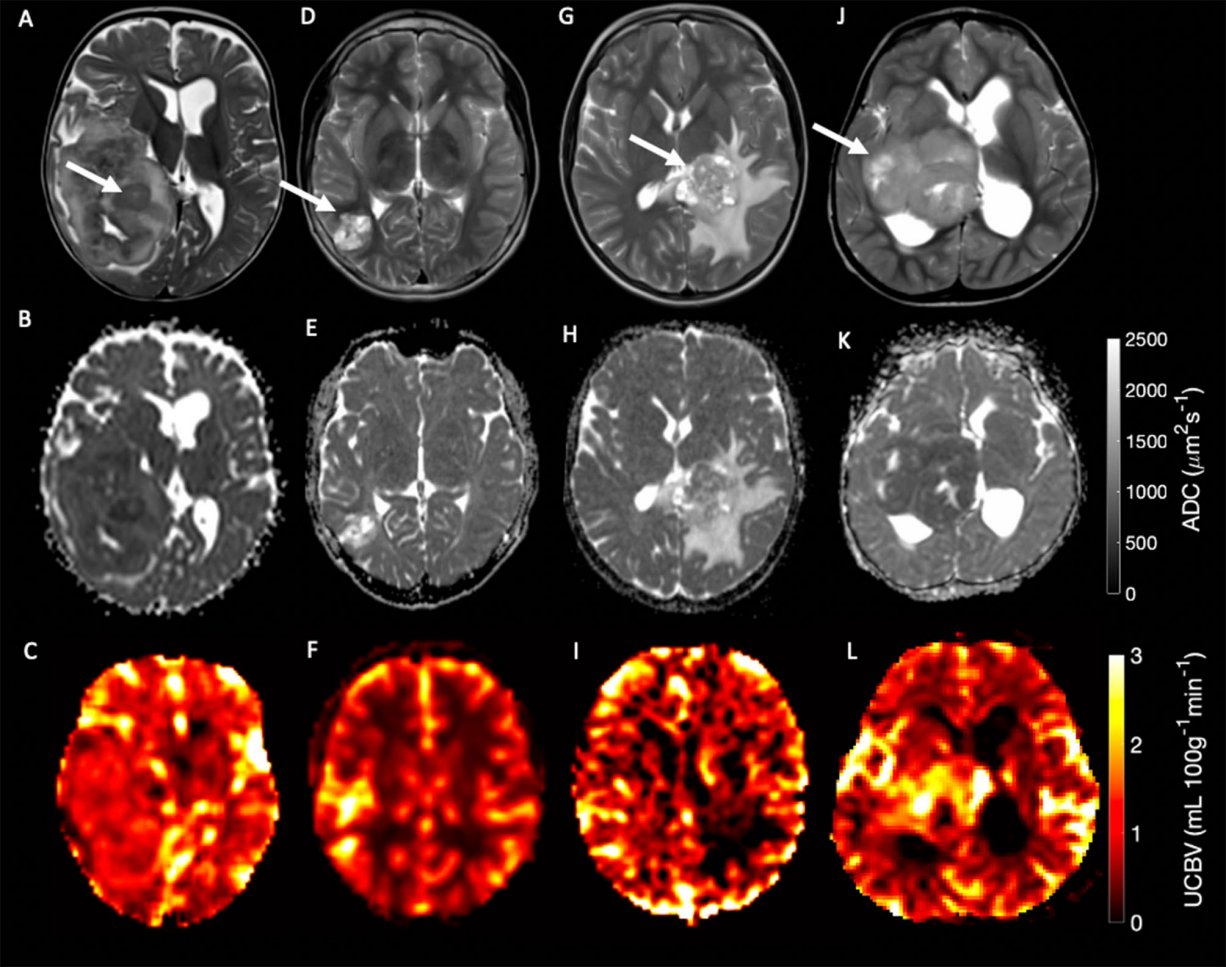
Example T_2_ weighted, diffusion, and perfusion imaging of Ependymoma (**A**–**C** respectively), Pilocytic astrocytoma (**D**–**F**, respectively), choroid plexus carcinoma (**F**–**H** respectively) and a Glioblastoma (**I**–**K**, respectively). Tumor regions are highlighted with white arrows.

**Figure 3:**
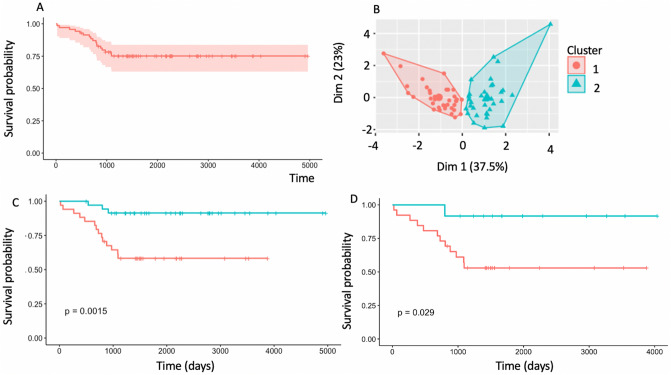
(**A**) Overall survival curve for the cohort, (**B**) K Means clustering survival results showing two distinct clusters, (**C**) Kaplan–Meier curve for the two clusters showing a significant difference in survival, 1 = High risk, 2 = Low risk, (**D**) Kaplan–Meier curves for high-grade low-risk (green) and high-risk (red) patients showing a significant difference in survival from imaging at diagnosis.

A total of 69 patients were analyzed in this study with 33 imaging features, including tumor volume, derived per patient. Example tumor anatomical, diffusion, and perfusion imaging can be seen in Fig. 2. The survival curve for the whole cohort is seen in Fig. 3A, showing 75% overall survival at 5 years from diagnosis.

### Diffusion and perfusion imaging can detect differences between tumor grade

Univariate statistical analysis showed significant differences in both whole brain and ROI imaging features between all high and low-grade tumors (feature with highest AUC = ADC mean (0.82) range: 0.63–0.82) full results detailed in supplementary Table 2. Comparative normal appearing grey and white matter imaging results are detailed in Supplementary Tables 3.

### Perfusion imaging plays a key role in assessing survival in pediatric brain tumors

Whole cohort univariate cox regression revealed a number of imaging features with significantly elevated hazard ratios (HR), for example Uncorrected CBV ROI mean (HR 3.1, Confidence Intervals (CI) 1.5–6.6, p = 0.003), full results detailed in Table 1.

**Table 1:** Cox regression results.

Feature	Beta	Hazard ratio	95% confidence interval	Significance
CBV ROI uncorrected mean	1.13	3.1	1.5–6.6	p = 0.003
CBV uncorrected standard deviation	− 1.12	0.33	0.11–0.99	p = 0.05
K2 ROI mean	− 2.02	0.13	0.03–0.63	p = 0.011
CBV uncorrected whole brain mean	1.12	3.02	1.06–8.91	p = 0.04

Bayesian analysis revealed the 5 most likely features to predict survival (probability that the feature coefficient is greater than 0, posterior coefficient) to be uCBV ROI mean (96%, 0.85), K2 ROI mean (39%, -0.17), uCBV whole brain mean (40%, 0.3), tumor volume (27%, 0.05), and ADC ROI kurtosis (20%, 0.02). Full results detailed in Table 2.

**Table 2:** Bayesian survival results.

Feature	Probability (%)	Posterior coefficient
Tumor volume	27	0.05
CBV ROI uncorrected mean	96	0.85
K2 ROI mean	39	− 0.17
ADC ROI Kurtosis	20	0.02
CBV uncorrected WB mean	40	0.3

### Unsupervised clustering detects distinct groups with significantly different survival and imaging characteristics

Using the Bayesian imaging features, k means clustering revealed two distinct clusters, shown in Fig. 3B, which when combined with Kaplan–Meier analysis revealed a significant difference between a high and low risk population (see Fig. 3C, p = 0.0015—overall survival for high and low risk = 55% and 90%, respectively). Cox regression revealed an elevated Hazard Ratio (HR 5.6, confidence intervals 1.6–20.1, p < 0.001) for the high-risk cluster, relative to the low-risk cluster.

Further univariate analysis of each cluster showed significant differences in a number of imaging features, for example elevated ADC kurtosis in high vs low risk clusters (10.1 ± 5.3 vs 4.3 ± 1.8, p < 0.001, respectively). A combination of both high- and low-grade tumors were found in both clusters, all other results detailed in Table 3.

**Table 3:** Low and high-risk cluster group features.

Feature	Low risk	High risk	Signficance
Male: female	16:19	19:15	N/A
Low: high grade	23:12	7:27	N/A
Censored: events	32:3	20:14	N/A
Tumor volume (cm^3^)	2.3 + 2.8	5.6 ± 7.0	p = 0.015
ROI ADC Kurtosis	4.3 + 1.8	10.1 + 5.3	p < 0.001
ROI ADC Skewness	0.1 ± 1.0	2.1 ± 1.0	p < 0.001
ROI K2 mean (min^−1^)	0.0018 ± 0.0027	− 0.005 ± 0.002	p < 0.001
ROI CBV uncorrected standard deviation (mL 100 g^−1^ min^−1^)	1.44 + 0.74	0.88 ± 0.42	p < 0.001
K2 whole brain standard deviation (min^−1^)	0.03 ± 0.02	0.019 + 0.008	p = 0.007
CBV corrected whole brain mean (mL 100 g^−1^ min^−1^)	1.14 + 0.27	1.29 ± 0.26	p < 0.02

The patient characteristics, split by risk cluster, is provided in Supplementary document 1. From this data, there was no significant difference between the low- and high-risk clusters in age at diagnosis (low-risk cluster mean age 7.3 years, high-risk cluster mean age 9 years, Mann–Whitney test p > 0.05), extent of surgical resection (complete macroscopic resection 18 cases in low-risk cluster, 19 cases in high-risk cluster, chi squared test, p > 0.05) or presence of metastatic disease (low-risk: focal 21 versus metastatic 7; high-risk: 18 focal versus 11 metastatic; chi squared test p > 0.05). However, the low-risk cluster contained a greater proportion of low-grade tumors (23 low-grade, 10 high-grade) than the high-risk cluster (7 low-grade; 26 high-grade), chi squared test p < 0.05. Whilst detailed data was not collected on adjuvant treatment with radiotherapy and chemotherapy, national treatment guidelines would have dictated that the high-risk cluster would in general have received more intense treatment regimens due to their generally higher grade.

### Supervised machine learning can be used to distinguish between high/low risk clusters

Supervised machine learning using imaging features showed that the Bayesian features combined with a single layer neural network, after stratified tenfold cross validation, provided the most accurate classification of high- and low-risk patients (accuracy = 98%, F-statistic = 0.98). Classifier accuracy ranged from 90 (logistic regression) to 98% (single layer neural network).

### There is a distinct difference in survival between high- and low- risk high-grade tumors

**Figure 4:**
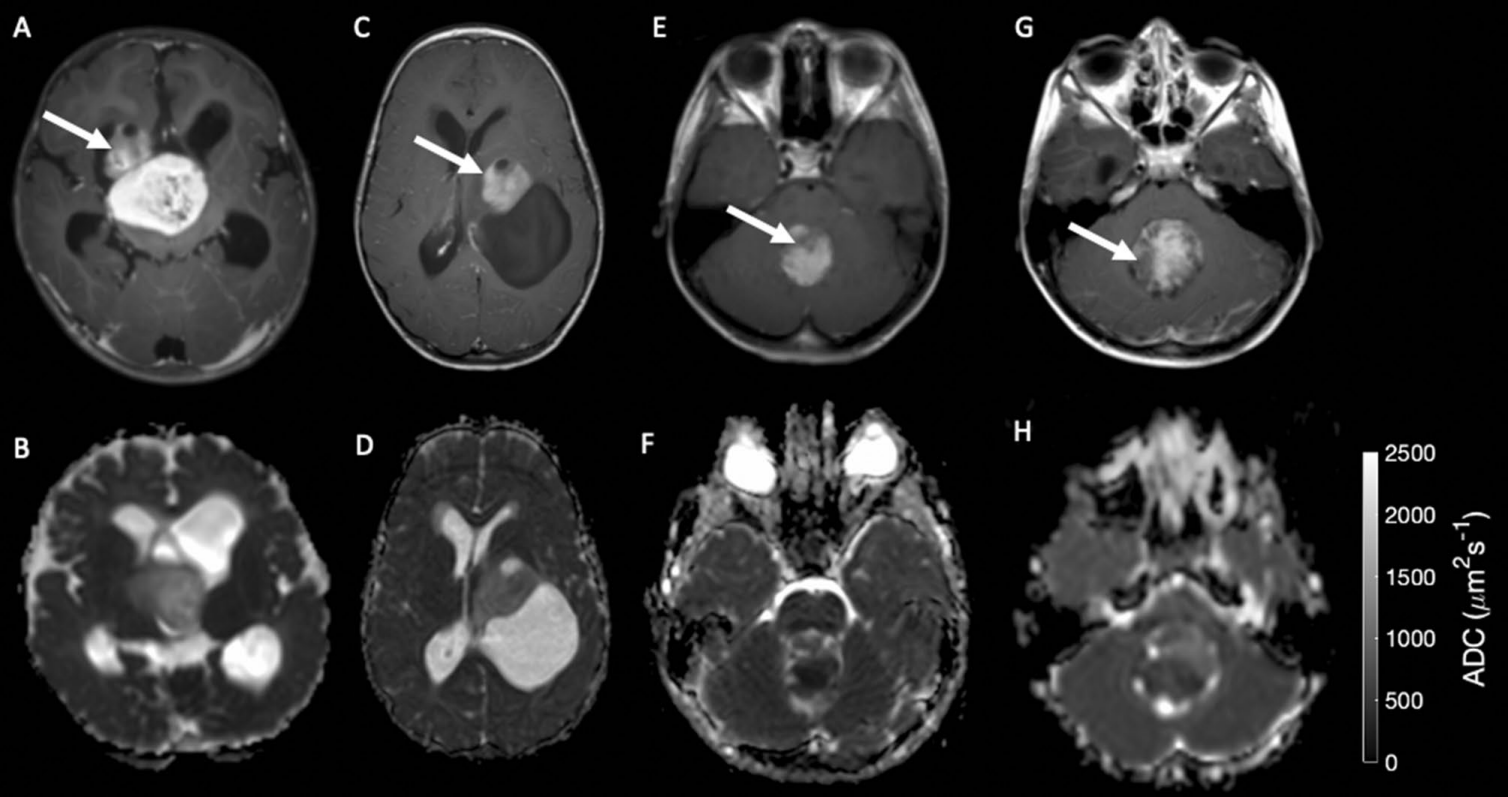
Example high and low risk, high and low-grade tumors. (**A** T1 post contrast & **B** ADC map) high risk and (**C** T1 post contrast & **D** ADC map) low risk Pilocytic astrocytoma, respectively showing elevated ADC skew and kurtosis in the tumor region. (**E**, **F**) high risk and (**G**, **H**) low risk medulloblastomas, respectively, showing increased ADC kurtosis. Tumor regions are highlighted with white arrows.

Kaplan–Meier analysis of high-grade tumors in the high and low risk clusters revealed a significant difference in survival (p < 0.05) with a hazard ratio of 7 (0.9–53 lower and upper bounds, respectively). The Kaplan–Meier curves for high grade tumors in both clusters can be seen in Fig. 3D. There was no detectable difference in survival between the high- and low- risk groups within the low-grade tumors (p > 0.05). Imaging of example cases by risk and grade given in Fig. 4.

Qualitative sub-group analysis of histology and genetics between low- and high-risk medulloblastomas revealed no significant differences between MYC amplification or groupings. The high-risk cluster exhibited a trend toward having a larger number of high Chang stage Medulloblastomas (M3 = 6, M2 = 4, M1 = 3) in comparison to the low-risk cluster (M2 = 1, M1 = 1, M0 = 2)—data shown in supplementary document 1.

## Discussion

This study has shown the power of combining diffusion and perfusion imaging with machine learning to predict survival risk in a mixed cohort of pediatric brain tumors. A handful of studies have previously looked at assessing survival with one of the aforementioned imaging techniques^14,16,27^; however, here we have shown the utility of combined diffusion-perfusion measures to provide advanced modelling of survival. The univariate results assessing low/high grade suggested a number of key diffusion and perfusion features for the discrimination between groups, however most had a poor AUC. Therefore, this represented an ideal situation for the use of machine learning to combine these features to provide highly accurate classifiers to solve this challenge.

Interestingly, the majority of parameters predicting survival were from the perfusion imaging which is not currently part of routine clinical practice in many centers. DWI has become a standard method for investigating childhood brain tumors and low ADC is seen as being a marker for higher cellularity and grade which would be associated with poorer survival. The current study substantiates this but shows that DSC-MRI may be an even better modality for predicting survival. The importance of the vessel leakiness parameter K in survival prediction also implies that DSC-MRI may have advantages in survival prediction beyond that available from methods which do not include the injection of contrast agent such as ASL. Furthermore, for medulloblastomas, clustering demonstrated a reasonable separation of high (M1 to M3) from low (M0) Chang stage^28^ tumors suggesting that these imaging features identify some properties in the primary tumor which are associated with metastatic potential.

There are a number of clinical risk factors which are commonly used to stratify treatment and it is important to consider the new imaging risk classification with these. The size of the cohort and relatively low number of events precluded a formal survival analysis with multiple risk factors. However, there was no significant difference in age, presence of metastatic disease or complete resection rate between the high- and low-risk imaging groups implying that the new imaging risk stratification will add value to these well-known risk factors. There was a significantly greater proportion of high-grade tumors in the high-risk imaging group as we would expect but the split is far from complete and a separate analysis of high-grade tumors showed a difference in survival between the high- and low-risk imaging groups showing added value beyond grade. Survival will be affected by treatment received and we did not systematically acquire information on radiotherapy and chemotherapy. However, on average the higher-risk imaging group will have received more intense treatment due to its greater proportion of high-grade tumors and so whilst adjuvant treatment is a confounder in the survival analysis, it will have acted to reduce the effect size thereby further increasing confidence in the robustness of the imaging risk classification.

The unsupervised machine learning identified two groups of tumors which did not correspond to any obvious non-imaging tumor characteristics. The credibility of these groups as being distinct entities was substantiated by the high accuracy (up to 98% on cross validation) with which the tumors could be assigned to the correct group by a supervised learner. A number of patients in the high-risk cluster were still alive at the study end although some of these, including those from known poor prognostic groups had short follow-up times. Further analysis showed that a number of surviving high-risk low-grade tumors had imaging features similar to high grade tumors (such as elevated ADC kurtosis and CBV) and were significantly different to low-risk low grade tumors. It will be interesting to ascertain the clinical course of these tumors over longer periods of follow-up. Interestingly, it is noted that survival risk status was not always associated with tumor grade (for example grade 3 vs 4) or histology, and this may point to further underlying tumor heterogeneity within current groupings.

A particular strength of this work is that imaging features with clinical data provide a non-invasive tool that can assess risk early in the patient journey. Indeed, the use of a supervised classifier to predict risk category allows for the prospective integration of this model into a clinical decision support system—whereby radiological analysis of a small number of imaging features can rapidly identify patients that should be considered for inclusion into clinical trials for prospective evaluation and subsequent stratification. The use of in vivo imaging also has the advantage that it provides information that cannot be found from analysis of resected tissue, perfusion in particular is inherently an in vivo property.

A further strength of this study is the use of multi-site, multi-scanner data—providing reassurance that the results are robust to the natural variability that occurs in protocols and scanners within clinical practice. Using multiple centers also provided a more statistically powerful study from which clinically relevant results could be obtained.

The imaging modalities used in this study are widely available and so data acquisition should be readily achieved in routine clinical practice. The image processing and classification should be made available by integration into a clinical decision support tool which are increasingly being developed^29^. Indeed, the results shown above show that it is possible to stratify patients into high and low risk groups with a trained supervised neural network, therefore enabling further real-time decisions to be made with regards to appropriate clinical management and inclusion into research trials for novel therapies to aid those with the current worst prognosis.

With the current uncertainty surrounding the use of Gadolinium in clinical practice, and the inability to be used in patients with impaired renal function, future work will include the addition of ASL^12^, a technique to estimate perfusion without the introduction of exogenous contrast agents, as data from this technique has been shown to correlate well with DSC cerebral blood volume^30,31^.

The main limitation of this study is that it is based on a relatively small heterogeneous cohort treated in a diverse manner. The results should be verified in a larger prospective study but it would also be interesting to apply the same methodology to specific tumor cohorts treated in a designated manner as part of a clinical treatment trial. The multi-center nature of the study is a strength but presents particular challenges notably with regards to variations in scanner protocol which may introduce variability in results. To some extent this was mitigated by calculating the ADC values directly from the raw DWI data for all cases and performing a leakage correction on the DSC time courses to allow for differences in Gd contrast injection protocols. Regions of interest were drawn by hand which could lead to variability but a previous study in children’s brain tumors by our consortium has shown good inter-rater consistency for all the ADC histogram metrics^32^. Finally, it is noted that there are a number of children alive at study end with high-risk tumors and currently limited follow-up. For example, a Choroid Plexus Carcinoma with a current follow-up of 1 year and a national average 5-year survival rate of 26%^26^ and a medulloblastoma with less than 3-year follow-up and M3 Chang stage.

In conclusion, this work has demonstrated a highly novel clinical application of advanced survival modelling and machine learning to non-invasively stratify patients according to risk. This provides a non-invasive, multi-modal MRI approach to determining the malignant nature of a tumor and its potential for poor prognosis. Both diffusion and perfusion were found to be important in determining risk, with perfusion contributing to a greater extent emphasizing the importance of acquiring perfusion imaging. This work represents an important step forward in the use of machine learning to predict survival and paves the way for further clinical studies focusing on the successful identification and treatment of high-risk children with brain tumors.

## Supplementary Information

Supplementary Information 1.

Supplementary Information 2.
