# Supplementary material for: Combining multi-site magnetic resonance imaging with machine learning predicts survival in pediatric brain tumors
Source: Sci Rep. 2021 Sep 23;11:18897. doi: 10.1038/s41598-021-96189-8 (PMC8460620; doi:10.1038/s41598-021-96189-8)
Supplement: Supplementary file 1 — Supplementary Information 1. [file 41598_2021_96189_MOESM1_ESM.docx]

Combining multi-site Magnetic Resonance Imaging with machine learning predicts survival in pediatric brain tumors.

**Authors:** James T. Grist PhD^1^, Stephanie Withey PhD ^1,2,3^, Christopher Bennett PhD ^1^, Heather E. L. Rose PhD ^1,2^, Lesley MacPherson PhD ^4^, Adam Oates PhD ^4^, Stephen Powell MSc^1^, Jan Novak PhD ^2,5,6^, Laurence Abernethy PhD ^7^, Barry Pizer PhD ^8^, Simon Bailey PhD ^9^, Steven C. Clifford PhD^10^, Dipayan Mitra PhD ^11^, Theodoros N. Arvanitis PhD ^1,2,12^, Dorothee P. Auer PhD ^13,14^, Shivaram Avula FRCR ^7^, Richard Grundy PhD ^15^, Andrew C Peet PhD ^1,2^_._

1. Institute of Cancer and Genomic Sciences, School of Medical and Dental Sciences, University of Birmingham, Birmingham, UK.
2. Oncology, Birmingham Women’s and Children’s NHS foundation trust, Birmingham, United Kingdom.
3. RRPPS, University Hospitals Birmingham NHS foundation trust, Birmingham, United Kingdom.
4. Radiology, Birmingham Women’s and Children’s NHS foundation trust, Birmingham, United Kingdom.
5. Psychology, College of Health and Life Sciences Aston University, Birmingham, United Kingdom.
6. Aston Neuroscience Institute, Aston University, Birmingham, United Kingdom.
7. Radiology, Alder Hey Children’s NHS foundation trust, Liverpool, United Kingdom.
8. Oncology, Alder Hey Children’s NHS foundation trust, Liverpool, United Kingdom.
9. Sir James Spence Institute of Child Health, Royal Victoria Infirmary, Newcastle upon Tyne, United Kingdom.
10. Wolfson Childhood Cancer Research Centre, Newcastle University Centre for Cancer, University of Newcastle, United Kingdom.
11. Neuroradiology, Royal Victoria Infirmary, Newcastle Upon Tyne, United Kingdom.
12. Institute of Digital Healthcare, WMG, University of Warwick, Coventry, United Kingdom.
13. Sir Peter Mansfield Imaging Centre, University of Nottingham Biomedical Research Centre, Nottingham, United Kingdom.
14. NIHR Nottingham Biomedical Research Centre, Nottingham, United Kingdom.
15. The Children’s Brain Tumour Research Centre, University of Nottingham, Nottingham, United Kingdom.

Corresponding author: Professor Andrew Peet, a.peet@bham.ac.uk.

**Conflicts of interest**

There are no conflicts of interest to disclose.

**Funding**

We would like to acknowledge funding from The CCLG and Little Princess Trust (CCLGA 2017 15) who funded Dr James Grist, Action Medical Research and the Brain Tumour Charity (GN2181), Children with Cancer (15/188), Cancer Research UK and EPSRC Cancer Imaging Programme the Children’s Cancer and Leukaemia Group (CCLG) in association with the MRC and Department of Health (England) (C7809/A10342), the Cancer Research UK and NIHR Experimental Cancer Medicine Centre Paediatric Network (C8232/A25261), the Medical Research Council – Health Data Research UK Substantive Site and Help Harry Help Others charity. Professor Peet is funded through an NIHR Research Professorship, NIHR-RP-R2-12-019. Stephen Powell gratefully acknowledges financial support from EPSRC through a studentship from the Physical Sciences for Health Centre for Doctoral Training (EP/L016346/1). Theodoros Arvanitis is partially funded by the MRC (HDR UK). We would also like to acknowledge the MR radiographers at Birmingham Children’s Hospital, Alder Hey Children’s Hospital, the Royal Victoria Infirmary in Newcastle and Nottingham Children’s Hospital for scanning the patients in this study. We would also like to thank Selene Rowe at Nottingham University Hospitals NHS Trust for help with gaining MRI protocol information.

**Author contributions**

Study conception and design: AP, RG, BP, SB, SC

Data analysis: JG, SW

Manuscript writing: JG, AP

Manuscript reviewing: JG, SW, CB, HR, LM, BP, SB, SC, DM, TA, DA, SA, RG, AP, AO, SP, JN, LA

Supplementary methods

The following list describes the imaging features derived from ADC, DSC, and T_2_ weighted imaging:

ADC mean, skewness, kurtosis, and standard deviation. uCBV mean, skewness, kurtosis, and standard deviation. cCBV mean, skewness, kurtosis, and standard deviation. K2 mean, skewness, kurtosis, and standard deviation. Whole brain ADC mean, skewness, kurtosis, and standard deviation. Whole brain uCBV mean, skewness, kurtosis, and standard deviation. Whole brain cCBV mean, skewness, kurtosis, and standard deviation. Whole brain K2 mean, skewness, kurtosis, and standard deviation. Tumor volume

Supplementary figures and tables

**Supplementary Table S1 - Imaging parameters used in this study.**

**
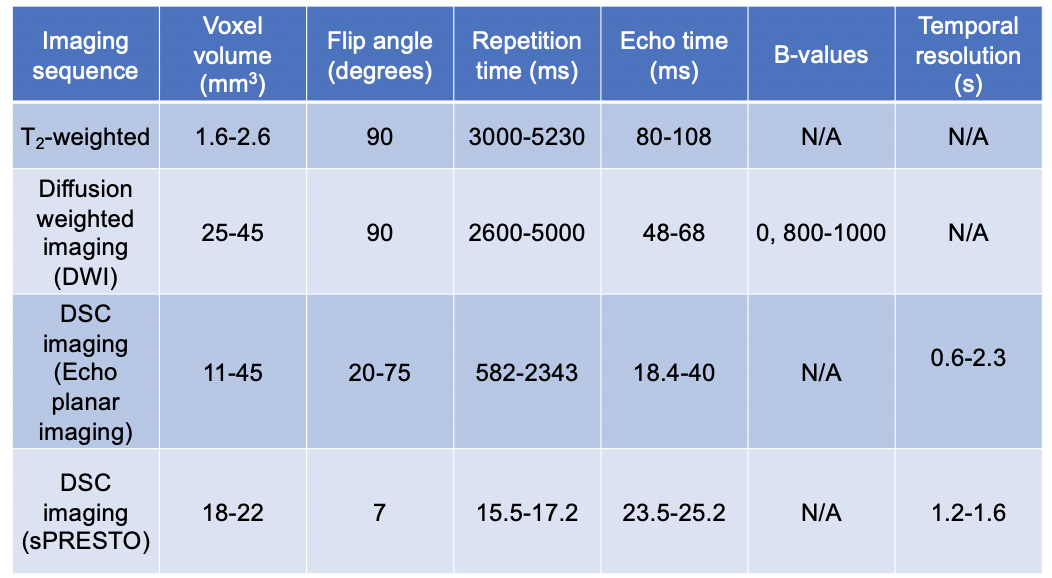
**

**Supplementary Table S2 - Differences in imaging features between high and low-grade tumors. (A) shows region of interest and (B) whole brain results. AUC = area under the curve.**

**
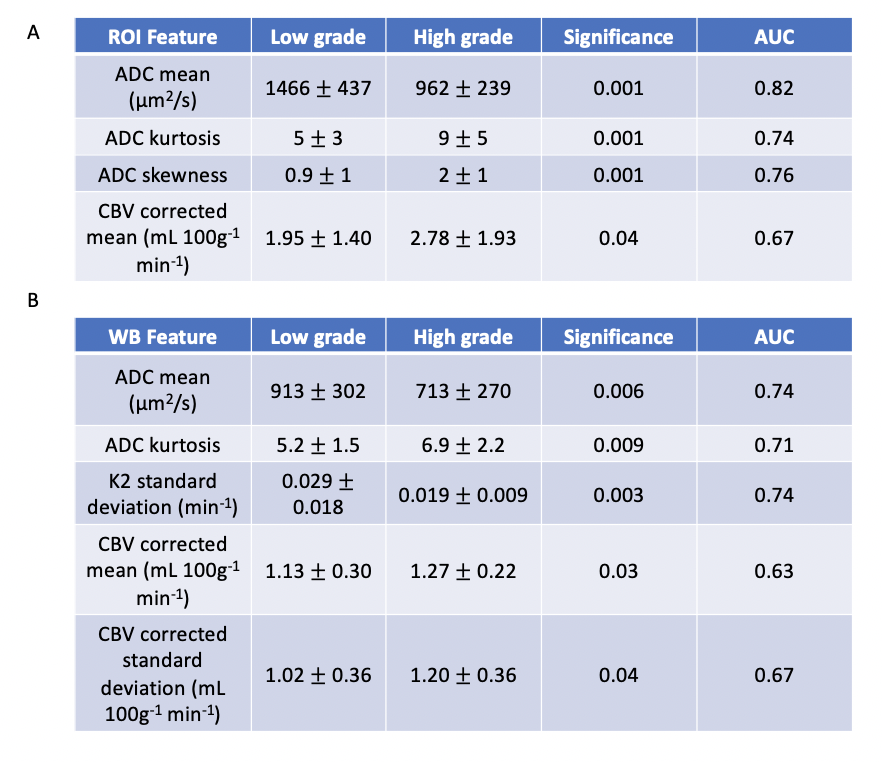
**

**Supplementary Table S3 - Average gray and white matter diffusion and perfusion values for the brain tumor cohort**

**
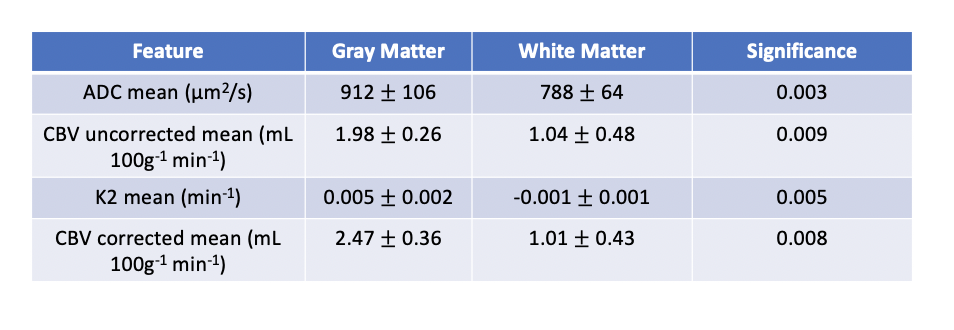
**
